# Reconstructing direct and indirect interactions in networked public goods game

**DOI:** 10.1038/srep30241

**Published:** 2016-07-22

**Authors:** Xiao Han, Zhesi Shen, Wen-Xu Wang, Ying-Cheng Lai, Celso Grebogi

**Affiliations:** 1 School of Systems Science, Beijing Normal University, Beijing, 100875, P. R. China; 2 Business School, University of Shanghai for Science and Technology, Shanghai 200093, P. R. China; 3 School of Electrical, Computer and Energy Engineering, Arizona State University, Tempe, Arizona 85287, USA; 4 Institute for Complex Systems and Mathematical Biology, Kings College, University of Aberdeen, Aberdeen AB24 3UE, UK

## Abstract

Network reconstruction is a fundamental problem for understanding many complex systems with unknown interaction structures. In many complex systems, there are indirect interactions between two individuals without immediate connection but with common neighbors. Despite recent advances in network reconstruction, we continue to lack an approach for reconstructing complex networks with indirect interactions. Here we introduce a two-step strategy to resolve the reconstruction problem, where in the first step, we recover both direct and indirect interactions by employing the Lasso to solve a sparse signal reconstruction problem, and in the second step, we use matrix transformation and optimization to distinguish between direct and indirect interactions. The network structure corresponding to direct interactions can be fully uncovered. We exploit the public goods game occurring on complex networks as a paradigm for characterizing indirect interactions and test our reconstruction approach. We find that high reconstruction accuracy can be achieved for both homogeneous and heterogeneous networks, and a number of empirical networks in spite of insufficient data measurement contaminated by noise. Although a general framework for reconstructing complex networks with arbitrary types of indirect interactions is yet lacking, our approach opens new routes to separate direct and indirect interactions in a representative complex system.

Network reconstruction, the inverse problem in complex networked systems, is of utmost importance in interdisciplinary fields^1^,^2^,^3^,^4^. The inverse problem is fundamental for understanding many social, biological and technological systems with complex interaction structures that are difficult or unable to be directly accessed. Typical examples include private relationship networks^5^,^6^, gene regulatory networks^7^,^8^, debit and credit networks among financial institutions and etc^9^. Scientific communities have increasingly recognized that a complex networked system should be explored as a whole rather than separate it into components to understand a variety of emergent phenomena^10^,^11^,^12^. Thus, network reconstruction from measurable data becomes the fundamental problem in the study of complex systems. Many approaches based on statistical physics, information theory and reverse engineering have been developed to address the problem, such as compressed sensing^3^,^5^,^6^,^45^, Pearson or Spearman correlation^13^, mutual information^14^, maximum entropy^15^,^16^ and Granger causality^17^. However, a significant challenge arises if there exists indirect interactions among nodes.

The scenario of indirect interaction is common, especially in social and economic systems, in which there may be indirect exchanges between two individuals without immediate connection but with common neighbors, such as a group of people to invest a joint project or buy the same stock^18^, some organizations participating in climate clubs to obey same rules and gain benefits together^19^, and the epidemic spreading among strangers because they participate in the party organized by their common friends^20^, etc. The effect of indirect interactions on individual states will be reflected in the measurable data in a complex manner. At present, most existent tools of network reconstruction are developed for the scenario without indirect interactions and we continue to lack an effective approach to distinguish between direct and indirect connections^13^,^14^,^15^,^16^,^17^.

Indirect interactions are typical in the public goods game (PGG) that has been a paradigm for exploring cooperative behaviors and social dilemmas in society and animal groups, such as global warming and economic inequality^21^,^22^,^23^,^24^,^25^,^26^,^27^,^28^,^29^,^30^,^31^,^32^,^33^. We aim to reconstruct networked PGGs with arbitrary topology from measurable individual data. In contrast to the networked two-player game, such as the prisoner’s dilemma game, snowdrift game and ultimatum game^5^,^6^, in the networked PGGs, players play the PGGs with not only their immediate neighbors but also their neighbors’ neighbors^23^. However, only direct interactions are associated with links among nodes, whereas indirect interactions are not. Thus, the key for achieving network reconstruction lies in distinguishing between direct and indirect interactions. We accomplish this goal by developing a two-step strategy. Firstly, we reconstruct a combined matrix composed of both direct and indirect interactions in terms of the mapping of the reconstruction problem into a sparse signal reconstruction problem and employ the Lasso to solve the problem^34^,^35^. Secondly, we identify all direct interactions (links) from the combined matrix by virtue of matrix transformation and optimization method. For homogeneous networks, full reconstruction can be achieved by using our method from a small amount of data. For heterogeneous networks, full reconstruction of networks can be achieved as well, but requires much larger amounts of data because of the existence of hubs. To better mimic the real situation, data measurement that is contaminated by noise has been used to implement reconstruction. We find that high reconstruction accuracy can be still achieved, demonstrating the robustness of our approaches against noise. Moreover, our method can be used to identify hidden node without any accessible information and reconstruct the connections among the rest nodes.

It is noteworthy that quite recently two methods, namely the silencing method and network deconvolution are proposed to separate direct correlation and indirect correlation to infer direct connections^36^,^37^. However, the indirect interactions are different from indirect correlation that naturally presents in any complex networks. Indirect correlation implies similar behavior between two disconnected nodes, whereas indirect interactions mean that there are physical interactions between two nodes without immediate connection. In other words, two nodes can indirectly interact with each other through a common neighbor of them in spite of the lacking of a direct connection between them. It is imperative to distinguish between direct and indirect interactions; otherwise, fake connections corresponding to indirect interactions will arise, accounting for the failure of network reconstruction. Although a general framework for reconstructing networked systems with arbitrary dynamics and any types of indirect interactions is still an open question at present, our work as the first attempt to deal with the PGG systems opens a route towards eventually resolve the problem in a comprehensive manner.

## Results

### Networked public goods games

**Figure 1:**
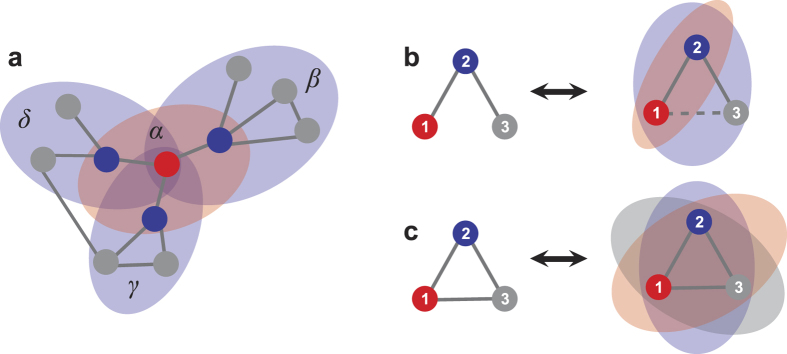
The schematic illustration of playing PGGs. (**a**) The focal player (red node) participates in 4 different groups of PGGs (*α*, *β*, *γ* and *δ*). Group *α* is centred on the focal individual and the other groups *β*, *γ* and *δ* are centered on the focal player’s neighbors, respectively. The payoff of the focal player stems from participating in four groups of PGGs. The solid links represent direct interactions among players. (**b**) The focal player 1 (red node) has one direct link with player 2 (blue node) and one indirect interaction (dashed line) with player 3 (gray node) due to their common neighbor (player 2). In this situation, the focal player 1 belongs to 2 different groups of PGGs (one self-centered PGG and the other PGG centered on player 2) and the payoff of player 1 will be affected by the action of player 3. (**c**) If the indirect interaction between player 1 and 3 in (**b**) is changed to a direct interaction, the focal player 1 will participate in 3 different groups of PGGs (one self-centered PGG and the other two groups centered on player 2 and 3, respectively), rendering the player 1’s payoff different from that in (**b**).

In the original PGG with *m* ≥ 2 players, at each round, every player is allocated with an endowment of *e* points, and is required to contribute *c*_*i*_(0 ≤ *c*_*i*_ ≤ *e*) points to a common pool. The total contribution is multiplied with an enhancement factor *b* (1 < *b* *<* *m*) and the result is distributed among all *m* members. Thus, the payoff of player *i* in the original PGG is 
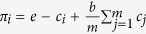
. In the networked PGGs, a player, say *i*, can take part in *k*_*i*_ + 1 original PGGs (one self-centered and *k*_*i*_ neighbor-centered) simultaneously, and contribute the same *c*_*i*_ to all *k*_*i*_ + 1 public pools. Thus, the total payoff of player *i* obtained from the networked PGGs at a given round *t* can be formulated as




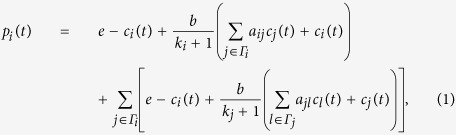




where Γ_*i*_ is the set of *i*’s neighbors and *N* is the number of players in the networked PGGs, adjacency matrix *A* ≡ {*a_ij_*}_*N×N*_ represents direct links among players (*a*_*ij*_ = 1 if *i* and *j* are connected and *a*_*ij*_ = 0 otherwise). In Eq. (1), the first term on the right-hand side is the payoff of participating in the self-centered PGG, and the second term is the payoffs of participating in *k*_*i*_ neighbor-centered PGGs. In the self-centered PGG, player *i* interacts with his/her direct neighbors, whereas in the neighbor-centered PGGs, player *i* interacts with not only his/her direct neighbors but also the neighbors’ neighbors, as shown in Fig. 1(a).

Figure 1(b,c) illustrate the need for distinguishing between direct and indirect interactions in the PGG. As shown in Fig. 1(b), the payoff of focal player 1 stems from two PGGs, one self-centered PGG and the other PGG centered on player 2. The indirect interaction (the dashed line) between player 1 and 3 stems from the fact that they both participate in the PGG centered on player 2 and their payoff is affected by the action of each other, although there is no direct link between them. It is necessary to accurately discern whether the interaction between player 1 and 3 is direct or indirect interactions; otherwise, if we fail to distinguish the interaction and treat the indirect interaction as a direct interaction (see Fig. 1(c)), the payoff of the focal player 1 will become quite different from the actual payoff. In this fake scenario, the focal player 1 participates in the self-centered PGG, the PGG centered on player 2 and the PGG centered on player 3, which apparently leads to a different payoff from the actual payoff of player 1 in Fig. 1(b). Thus, it is imperative to discern indirect interactions to successfully reconstruct network structure. The payoff difference between the actual scenario and the fake scenario offers sufficient information to achieve full reconstruction.

In the evolutionary PGGs, players are allowed to update their strategies by referring to their direct neighbors’ information according to, for example, the learn-from-best rule, random-selection rule, Fermi rule and so on. Here, without loss of generality, we choose the Fermi rule as our strategy-updating rule. The strategy updating probability *W*_*i*←*j*_ of player *i* is




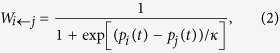




where player *i* choose his/her neighbor *j* randomly in *i*’s neighborhood and adopts player *j*’s strategy with probability *W*, *κ* is characterizes the stochastic uncertainties in the game dynamics. Indeed, when *κ* = 0, player *i* always adopts the strategy of the player with better payoff with probability 1, while as *κ* → ∞, player *i* updates his/her strategy with probability 1/2 regardless of the payoff difference. For simplicity, we set *κ* = 0.1 by following existent research in the literature^38^.

### Reconstructing combined matrix

As the first step of the two-step reconstruction strategy, we aim to reconstruct a combined matrix *C* consisting of both direct and indirect interactions. The key to the accomplish of the first step lies in the establishment of formula **Y**_*i*_ = Φ_*i*_ · **X**_*i*_ (*i* = 1, 2, …, *N*), in which vector **X**_*i*_ includes all direct and indirect interactions of node *i*, and vector ***Y***_*i*_ and matrix Φ_*i*_ can be constructed exclusively from the time series of individual payoff *p*_*i*_ and strategy *c*_*i*_.

To simplify our description of networked PGGs, we denote *G* ≡ *A* + *I*, where *I* is an identity matrix. Thus, Eq. (1) can be expressed as




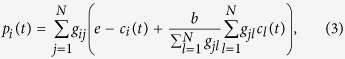




where 
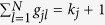
. The reconstruction formula can be established by focusing on the payoff of player *i*, determined by Eq. (3), which for convenience can be expressed in the matrix form (see Supplementary Note 1)




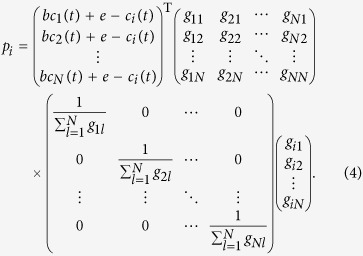




Let *s*_*ij*_ (*t*) = *bc*_*j*_ (*t*) + *e* − *c*_*i*_ (*t*), 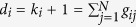 and




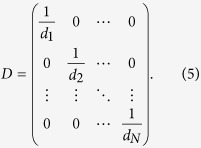




We can simplify the payoff of player *i* in an arbitrary round as follows




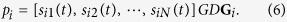




In the evolutionary PGG, all players play *M*-round games from *t*_1_ to *t*_*M*_ with their neighbors, providing sufficient information for building the reconstruction formula




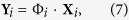




where matrix




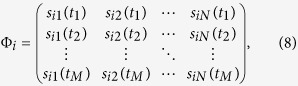




vector




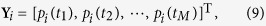




and vector




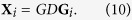




In the formula, matrix Φ_*i*_ and vector **Y**_*i*_ can be obtained from individual strategies and payoffs of players, respectively, allowing us to reconstruct **X**_*i*_ directly from Φ_*i*_ and **Y**_*i*_ by using the Lasso, a convex optimization method (see Methods for details). In a similar fashion, we can reconstruct vector **X**_*j*_ for node *j* and for all nodes as well, giving rise to the combined matrix




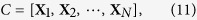




Note that *C* is similar to second-order transfer matrix^39^.

### Separation between direct and indirect interactions

Insofar as the combined matrix *C* is successfully inferred, we can distinguish between direct and indirect interactions in matrix *C*, giving rise to the adjacency matrix *A*, namely, the whole network structure. Specifically, according to Eqs. (10) and (11), we have




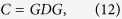




where *C* has been obtained and matrix *D* can be obtained in virtue of ∑ **X**_*i*_ = *d*_*i*_ (see Supplementary Note 2). Therefore, in principle, matrix *G* = *A* + *I* can be derived based on *C* and *D* and consequently, yields network structure *A*. However, deriving *G* directly from Eq. (12) is still challenging, calling for mathematical techniques and optimization to solve *G*.

First, we rewrite Eq. (12) to be




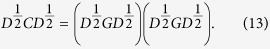




We perform similarity transformation on matrix 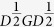, yielding




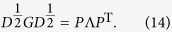




We can thus formulate




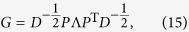




where *P*, Λ and *P*^T^ can be obtained by similarity transformation and the optimization based on the linear least squares method (see Methods for detailed derivation and optimization). Then the network matrix can be directly inferred via




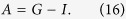




**Figure 2:**
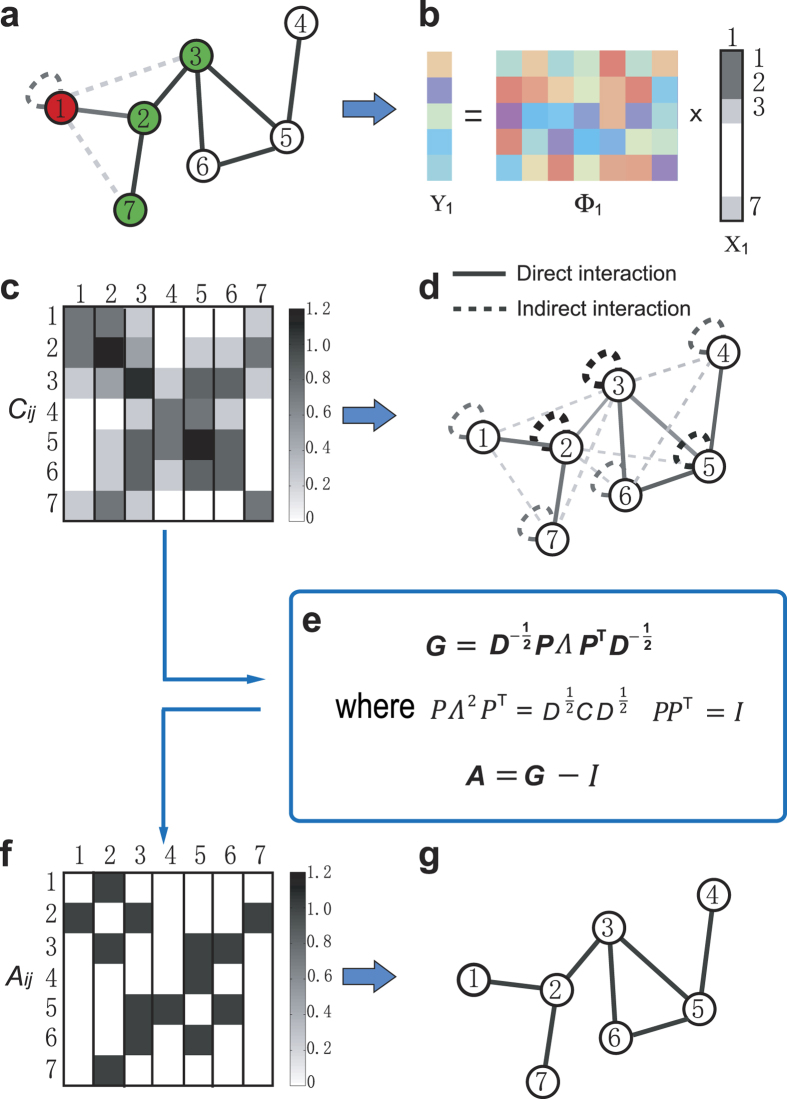
Illustration of reconstructing actual links. (**a**) The red node (node 1) has one direct interaction with node 2 (solid line), and indirect interactions with node 1 and node 3 (dashed lines). Node 1 has a virtual self-loop. The other nodes also have indirect interactions with their neighbors’ neighbors and virtual self-loops (for clarity, the indirect interactions and virtual self-loops of the other node are not shown in the figure). (**b**) We can build the relationships between the payoffs and strategies of the red node **Y**_1_ = Φ_1_ · **X**_1_ from data, where vector **X**_1_ contains all direct and indirect interactions with the red node. If we can decode the vector **X**_1_ accurately, the values in the first, second, third and seventh rows corresponding to interactions with nodes 1, 2, 3 and 7 will be nonzero, while the other values are zero. (**c**) In the same fashion, we can build the vector **X**_*i*_ of all nodes and comprise the combined matrix *C*. (**d**) The direct interactions (solid lines) and indirect interactions (dashed lines) can not be distinguished directly based on the network which is derived from the combined matrix *C*. (**e**) To distinguish between the direct interactions and indirect interactions, adjacency matrix is achieved through the two equations with similarity transformation and the linear least squares method. (**f**) Compared with combined matrix, the indirect interactions and self-loops are removed, and the nonzero elements in adjacency matrix denote actual links. (**g**) The indirect interactions are removed completely and the original network is recovered.

An intuitive illustration of our two-step reconstruction process is shown in Fig. 2. As shown in Fig. 2(a), node 1 (the red node) has one direct interaction (solid line) with node 2 and two indirect interactions (dashed lines) with node 3 and node 7. Moreover, node 1 has a virtual self-loop (dashed lines) due to intrinsic dynamics of the PGGs. In fact, all nodes interact with themselves, and the self-interaction captured by the virtual self-loop is subject to indirect interactions, because of the absence of the self-loop in the network structure. By using the Lasso to optimize the reconstruction formula (Fig. 2(b)), we can reconstruct both direct and indirect interactions of node 1, included in vector **X**_1_. By repeating the reconstruction process shown in Fig. 2(a,b), we can obtain the combined matrix *C* (Fig. 2(c)) that includes both direct and indirect interactions of all the nodes. As shown in Fig. 2(d), direct interactions (solid lines) and indirect interactions (dashed lines) cannot be distinguished in this stage. To separate the two types of interactions and identify direct links, we decompose the combined matrix *C* by exploiting similarity transformation and the linear least squares method to solve the Eq. (15) (Fig. 2(e)), allowing us to obtain adjacency matrix *A* (Fig. 2(f)) from matrix *C* eventually. Compared to Fig. 2(d), the indirect interactions captured by dashed lines are removed in Fig. 2(g), recovering the original network in Fig. 2(a) accurately.

### Network reconstruction performance

**Figure 3:**
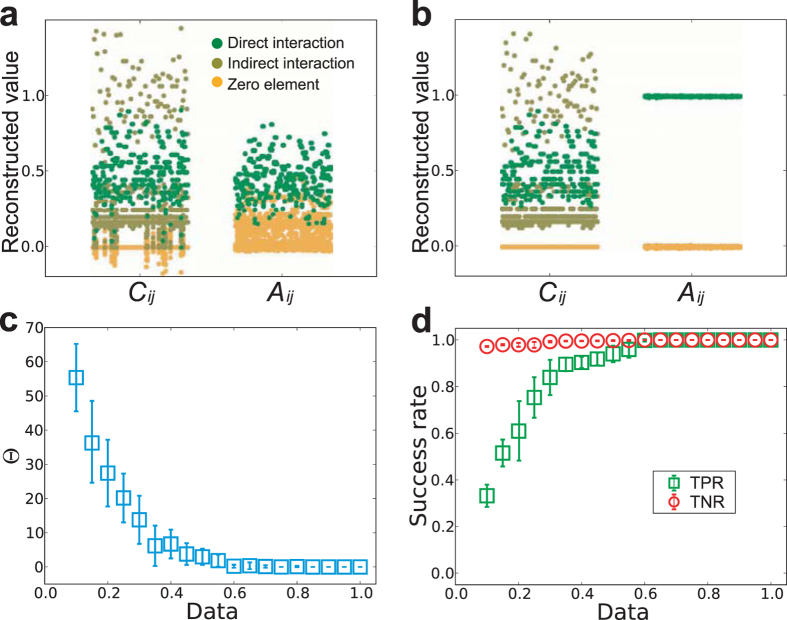
The performance of reconstructing WS small-world networks. (**a**) Reconstructed values of elements *C*_*ij*_ and *A*_*ij*_ with a small amount of data, Data = 0.4. (**b**) Reconstructed values of elements *C*_*ij*_ and *A*_*ij*_ with a relatively more data, Data = 0.6. (**c**) The data-based index Θ of measuring the precision of reconstructing combined matrix *C*. (**d**) Success rate of inferring WS networks based on time series of payoffs and strategies from the evolutionary PGGs. The network size is 100, and average degree 〈*k*〉 = 4. Rewiring probability of WS small-world networks is 0.3. Each data point in (**c**,**d**) is obtained by averaging over 10 independent realizations. The error bars denote the standard deviations. The parameter *λ* in the Lasso is set 10^−3^.

We numerically simulate the PGGs occurring on both homogeneous and heterogeneous networks, including Erdös-Rényi (ER) random networks^40^, Watts-Strogatz (WS) small-world networks^41^, Newman-Watts (NW) small-world networks^42^ and Barabási-Albert (BA) scale-free networks^43^, and several real networks. Without loss of generality, we set *b* = 1.5 and *e* = 10 in our simulations. Initially, each node is occupied by a player with a random contribution *c*_*i*_, where 0 ≤ *c*_*i*_ ≤ *e* is an integer for simplicity. In each round, all players engage in the PGGs simultaneously and update their strategies according to the Fermi rule (see Methods). The strategies and payoffs of all players are recorded for network reconstruction by employing our method. Different amounts of data (Data ≡ *M*/*N*) are considered, where *M* is the length of time series (the number of observable rounds) and *N* is the network size. As shown in Fig. 3(a), for a small amount of insufficient data, e.g., Data = 0.4, elements *C*_*ij*_ in the combined matrix *C* corresponding to direct interactions (actual links), indirect interactions and null elements (zeros in combined matrix) disperse in a wide range, rendering the complete separation of the three types of elements in *C* and the accurate identification of actual links impossible. By exploiting similarity transformation and the linear least squares method, we can eliminate indirect interactions, giving rise to reconstructed adjacency matrix *A*. However, because of insufficient amounts of data, *A*_*ij*_ in the reconstructed adjacency matrix *A* corresponding to direct interactions (actual links) and null connections (null elements) still overlap a little bit, demonstrating that a full reconstruction of network structure was not achieved yet. In contrast, for a relatively more data, e.g., Data = 0.6, as shown in Fig. 3(b), a vast gap arises between actual links and null elements in the reconstructed adjacency *A*, although the direct and indirect interactions are mixed in the reconstructed matrix *C*. Hence, full reconstruction of networks can be ensured by our method from sufficient amount of but sparse data that could be less than the network size *N*. Although direct and indirect interactions cannot be distinguished in matrix *C*, an accurate reconstruction of matrix *C* is the prerequisite for implementing similarity transformation and optimization to precisely reconstruct matrix *A*. In this regard, we introduce a data-based index to measure the precision of reconstructing matrix *C*. To be specific, we define 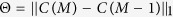, where 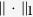 represents *L*_1_ entrywise norm and matrix *C*(*M*) is obtained with *M* time series. The fact that Θ approaches zero indicates that the reconstructed matrix *C* becomes stable and close to the actual *C* with small difference. As shown in Fig. 3(c), we see that as the amount of data increases, the value of Θ decreases rapidly. When the value of Θ is very small, e.g., Θ < 0.1, the gap between the two types of interactions and null elements in reconstructed *C* emerges (e.g., Fig. 3(b)), and thus matrix *C* can be considered to be accurately reconstructed. Insofar as *C* is accurately reconstructed, we can derive the degree of each node from *k*_*i*_ = ∑ **C**_*i*_ − 1. Consequently, the top *k*_*i*_ values of *i*’s row in adjacency matrix *A* can be regarded as actual links. The predicted node degree from *C* thus offers criterion for determining the neighborhood size of each node, without relying on an additional threshold to separate actual links from null connections for each node, and the use of the area under the receiver operating characteristic curve (AUROC) (see Supplementary Figure S1)^6^,^8^. To verify our method, we use the true positive rate (TPR) and true negative rate (TNR) as explicit indices to quantify the reconstruction performance, where TPR is defined as the average ratio of the number of successfully inferred links to the number of actual links for all nodes, and TNR is similarly defined for null elements in the adjacency matrix. If and only if both TPR and TNR reach 1, the network is said to be fully reconstructed. As shown in Fig. 3(d), for WS small-world networks, when the amount of data exceeds 0.3, 80% success rates for both TPR and TNR are achieved, which implies that most of links can be successfully inferred even for a quite small amount of data, e.g., Data=0.3. When Data exceeds 0.6, full reconstruction with 100% success rates is achieved from a small amount of data that are still less than the network size *N*.

Systemic reconstruction results for different types of networks, average degree 〈*k*〉 and variance of measurement noise *σ* are shown in Table 1. We see for all the considered cases, accurate reconstruction is achieved. In particular, for relatively large homogeneous networks, e.g., *N* = 500 for ER, WS and NW networks, only a small amount of data is required to ensure full reconstruction. However, compared with homogeneous networks, larger amounts of data are required for reconstructing heterogeneous networks, e.g., BA networks. For heterogeneous networks, there are hubs with much more neighbors compared with other nodes. The hubs immediately induce much more indirect interactions in their neighborhoods than that in homogeneous networks. As a result, the combined matrix *C* of heterogeneous networks is usually much denser than homogeneous networks. Note that the reconstruction method based on the Lasso needs less data for reconstructing a sparse vector X_*i*_. Thus, the much denser matrix *C* in heterogeneous networks accounts for the requirement of a larger amount of data to fully reconstruct heterogeneous networks by using the Lasso. In the presence of small measurement noise, e.g., 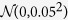, full reconstruction can be achieved by using slightly larger amounts of data compared to the results without noise, as displayed in Table 1. When data are contaminated by strong noise, e.g., 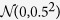, we can still reconstruct networks quite accurately from a relatively large amount of data for both homogeneous and heterogenous networks. These results suggest that our method is of both high efficiency and strong robustness against noise for reconstructing complex networks with indirect interactions. Table 2 shows the performance of our method in reconstructing several real social networks. We see that precise reconstruction can be achieved for all the real-world networks, which offers additional evidence for the practical applicability of our reconstruction approach.

### Identifying the hidden node

In the real situation, we may miss the information of some nodes because the nodes are inherently inaccessible or we are not aware of their existence. We explore the robustness of our reconstruction method against the presence of such hidden nodes to test the applicability of our method. Specifically, we assume a hidden node whose strategies and payoffs can not be recorded exists in the network, and our purpose it to identify the nodes who interact with the hidden node and reconstruct the rest nodes and their connections.

**Figure 4:**
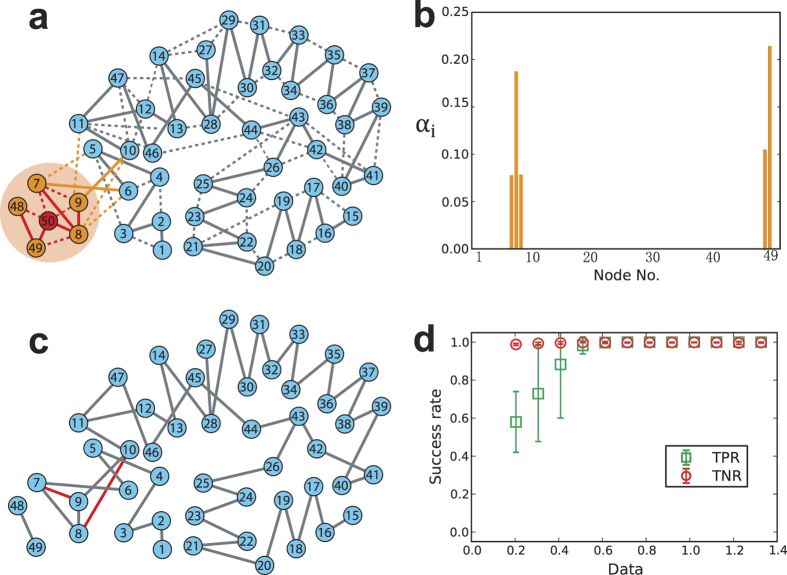
Identifying the hidden node and reconstructing the connections among the other nodes. (**a**) Illustration of a hidden node. The hidden node (node 50 in red) interacts with five nodes (in orange), including direct interactions (solid lines) and indirect interactions (dashed lines). For clarity, the virtual self-loop of each node and interaction weights are not shown. The direct (red solid lines) and indirect (red dashed lines) interactions between the hidden node and its neighbors and neighbors’ neighbors can be inferred in terms of the standard variance (Equation (17)). However, the direct and indirect interactions cannot be distinguished because of missing the data of the hidden node. The time series of the other nodes except the hidden node are measurable. The interactions (orange lines) between the neighbors of the hidden node and the other nodes except the hidden node can be reconstructed accurately by simply ignoring the hidden node. However, indirect (orange dashed lines) and direct interactions (orange solid lines) still cannot be distinguished. The other connections (gray lines) can be accurately reconstructed and classified. (**b**) The standard variance of reconstructed interactions *α*_*i*_ of each node. The five nodes who interact with the hidden node exhibits much larger values of *α* than the other nodes. (**c**) The reconstructed network except the hidden node. The gray lines are true existent links which are reconstructed successfully, and the red lines are false positive (fake) links which do not exist in the original network. (**d**) The success rate of reconstructing the WS small-world network in (**c**). In the WS small-world network, the network size *N* is 50 (including the hidden node), the average degree 〈*k*〉 = 2 and the rewiring probability is 0.1. The results are obtained by averaging over 10 independent realizations.

The basic idea of ascertaining and identifying the hidden node is based on missing information from the hidden node when attempting to reconstruct direct and indirect interactions of the network by using the first step in our reconstruction framework. In particular, to reconstruct the direct and indirect interactions belonging to the hidden node accurately, time series from the hidden node are needed to generate the matrix Φ_*i*_ and the vector **Y**_*i*_. However, no time series from the hidden node are available, leading to reconstruction inaccuracy and, consequently, anomalies in the predicted interaction patterns of the nodes who interact with the hidden node. It is then possible to detect the nodes who interact with the hidden node by identifying any abnormal interaction patterns^3^,^44^,^45^, which can be accomplished by using different data segments. If the inferred interactions of a node are stable with respect to different data segments, the node can be deemed to have no interaction with the hidden node; otherwise, if the result of inferring a node’s interactions varies significantly with respect to different data segments, the node is likely to interact with the hidden node. To identify the nodes who interact with the hidden node, we can define the standard variance of predicted interactions with respect to different data segments as follows:




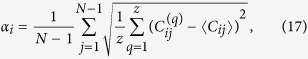




where 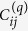 denotes the element value in the combined matrix *C* of the rest nodes inferred from the *q*th group of the data, 
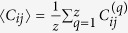
 is the mean value of *C*_*ij*_, *N* is the network size, and *z* is the number of data segments. Figure 4(a) illustrates that a hidden node interacts with five nodes in the exemplified network. Applying Eq. (17) to the reconstructed combined matrices yields the results shown in Fig. 4 (b), where the values of *α*_*i*_ associated with the nodes who interact with the hidden node are much larger than those of the other nodes (that are essentially zero).

In the presence of a small number hidden nodes, their influence of missing their data to the reconstruction of the whole network is negligible. Thus, we can directly using the two-step reconstruction method by ignoring the hidden node to reconstruct the connections among the nodes other than the hidden node. As shown in Fig. 4(c), all true existent links are inferred successfully. However, there are two redundant (fake) links (marked as red) that stems from the influence of the hidden nod. In Fig. 4(d), as the measurable data increase, the TPR and TNR of reconstructing the connections among the rest nodes approach unit, indicating that the network can be reconstructed quite accurately in spite of the existence of a hidden node in the network. The reconstruction performances for different types of networks with hidden nodes are shown in Table 1. It is worth noting that in the presence of a large fraction of hidden nodes, ignoring the influence of the hidden nodes will lead to many fake links, accounting for the failure of directly using the reconstruction method. At present, how to tackle a network with an arbitrary fraction of hidden node is still an extremely challenging question.

## Discussion

We have developed an approach with a two-step strategy to reconstruct interaction structure in networked PGGs with indirect interactions among nodes from measurable time series of individual strategies and payoffs. We first reconstruct both direct and indirect interactions among nodes by transferring the network reconstruction problem into a sparse signal reconstruction problem, which is solved by using the Lasso in an efficient and robust manner. Subsequently, we distinguish between direct and indirect links by virtue of similarity transformation and an optimization method based on the linear least squares. Moreover, our framework is able to locate hidden node and reconstruct the network structure except the hidden node. We have validated our method in terms of both homogeneous and heterogeneous networks, finding that high reconstruction accuracy can be achieved for all the studied cases. In general, less amounts of data are required for reconstructing homogeneous networks than that for heterogeneous networks, due to the existence of hubs. Our method is also resilient to measurement noise and hidden nodes, accounting for its practical importance in real networked systems.

Our work also raises a number of questions, answers to which could improve our ability to reconstruct complex networks with indirect interactions. First, with respect to a variety of indirect interactions in the real world, our approach cannot offer a general solution to the reconstruction problem at present, and only complex systems characterized by networked PGGs can be reconstructed by our framework, prompting us to wonder if a general reconstruction framework can be developed for complex networks with any indirect interactions? Second, relatively large amounts of data are required to reconstruct heterogeneous networks. A practically significant question is how can we reduce the data amount based on the current method? Third, although we can locate a hidden node by identifying all its interactions, how to distinguish direct and indirect interactions between the hidden node and the other nodes accurately and how to locate a large fraction of hidden nodes are still open questions. Fourth, the empirical test of our method is still lacking in spite of our systematic numerical investigations. Our approach is expected to be available in laboratory experiments of the networked PGGs by recruiting subjects. From the information of subjects in the game, the network structure can be reconstructed accurately. Analogously, our method is also applicable to networked climate game experiments^25^ that is closely related with the PGGs. Since usually direct and indirect interactions play a joint role in the dynamics of the whole system and their effects are hidden in measurable data in a complicated manner, we anticipate that it is challenging to answer these questions. Nonetheless, our approach as the first attempt opens a new route to solve the difficult problem with implications for understanding many social networked systems in a wide range of fields.

## Methods

### The Lasso for sparse signal reconstruction

The Lasso is a convex optimization method to reconstruct a vector **X**_*i*_∈*R*^*N*^ from linear measurements **Y**_*i*_ and Φ_*i*_ in the form




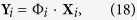




where **Y**_*i*_ ∈ *R*^*N*^ and Φ_*i*_ is an *M* × *N* matrix. **X**_i_ can be reconstructed by applying the Lasso for solving




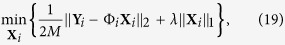




where *λ* is a nonnegative regularization parameter. The sparsity of solution is assured by 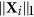 in the Lasso according to the compressed sensing theory^46^. Meanwhile, the least square term 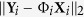 makes the solution more robust against noise in time series than *L*_1_-norm-based optimization method.

### Identification of direct and indirect interactions from combined matrix *C*

Notice that 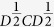 and 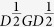 are both real symmetric matrices. Matrix 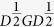 can be decomposed to be 
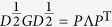
 via similarity transformation where Λ = diag(*λ*_1_, *λ*_2_, …, *λ*_*n*_), and *P* is an *N* × *N* nonsingular orthogonal matrix, which can be derived from similarity transformation of 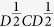, in which 
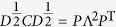
. Thus, the equation 
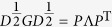
 can be expanded as follows




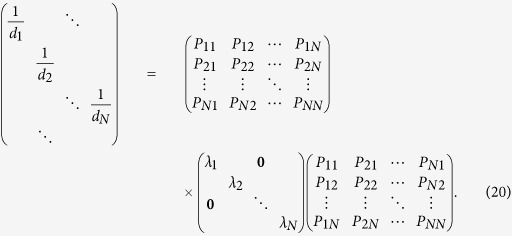




Although we can not ascertain each element in matrix 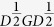 in this stage, we can still obtain diagonal elements 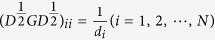 according to *d*_*i*_ = ∑ **C**_*i*_. Thus according to the Eq. (20), we obtain




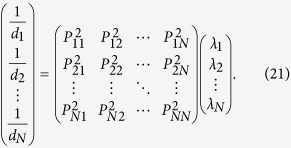




If 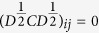, in correspondence with the element 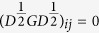, then we can extend the Eq. (21) as




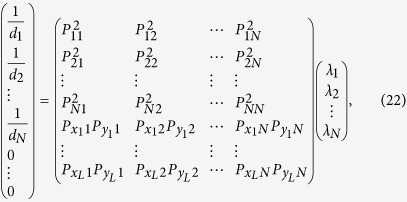




where *L* is the number of zeros in matrix 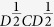. The above equation also satisfy the formation ***β*** = Ψ · ***α***, in which ***α*** = (*λ*_1_, *λ*_2_, 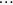, *λ*_*N*_)^T^, 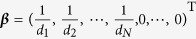, and Ψ is the corresponding matrix. In this situation, most of values in ***α*** are not zero, thus we can obtain ***α*** via the linear least squares method




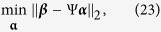




where vector ***α*** ∈ *R*^*N*^ from vector ***β*** ∈ *R*^*K*^ and matrix Ψ^(*N*+*L*)×*N*^. The optimization also known as *L*_2_ norm minimization, a basic optimization paradigm for solving an overdetermined system of linear equations. Due to the sparsity of adjacency matrix, the combined matrix *C* also contains lots of zeros, leading to 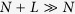 in the matrix Ψ, thus Eq. (22) can be well solved by the linear least squares method. Now we can get Λ via this method, then get 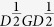 via 
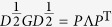
, and obtain Eq. (15).

## Additional Information

**How to cite this article**: Han, X. *et al*. Reconstructing direct and indirect interactions in networked public goods game. *Sci. Rep.* **6**, 30241; doi: 10.1038/srep30241 (2016).

## Supplementary Material

Supplementary Information

## Figures and Tables

**Table 1:** The performance of reconstructing different types of artificial networks.

*N*	〈*k*〉	*σ*	*N*_h_	ER	WS	NW	BA
	4	0	0	0.61	0.41	0.50	0.95
	4	0.05	0	0.64	0.48	0.49	1.20
100	4	0.5	0	1.35	1.80	1.74	5.02
	6	0	0	0.89	0.75	0.77	1.10
	8	0	0	1.06	0.97	1.03	1.15
300	4	0	0	0.24	0.2	0.25	0.71
500	4	0	0	0.20	0.15	0.19	0.46
100	4	0	1	1.81	0.77	0.51	1.51

Data amount needs to achieve 90% success rates (SR) for four artificial network models, where SR = TPR × TNR (SR is area under ROC with given threshold, see Supplementary Figure S1 for more details). ER, WS, NW and BA networks with different network size *N*, average degree 〈*k*〉 and measurement noise (Gaussian white noise 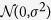) are considered. *N*_h_ denotes the number of the hidden nodes. The results are obtained by averaging over 10 independent realizations. More details of the success rates as a function of data amount for different cases can be found in Supplementary Figures S2–S5.

**Table 2:** The performance of reconstructing real social networks.

Networks	*N*	〈*k*〉	Data
Karate	34	4.6	1.17
Dolphins	62	5.1	0.76
Football	115	10.7	1.14
Santa Fe	118	3.4	0.46
Jazz	198	27.7	0.97
Email	1133	9.6	0.82

Minimum data for achieving at least 90% success rates (SR) for several real networks. The variables represent the same meanings as Table 1. More details of the real networks can be found in Supplementary Figure S6 and Supplementary Table S1.
