# Supplementary material for: Reconstructing direct and indirect interactions in networked public goods game
Source: Sci Rep. 2016 Jul 22;6:30241. doi: 10.1038/srep30241 (PMC4996070; doi:10.1038/srep30241)
Supplement: Supplementary Information [file srep30241-s1.pdf]

Supplementary Materials for

Reconstructing direct and indirect interactions in networked public goods game

Xiao Han[1], Zhesi Shen[1], Wen-Xu Wang*[1,2], Ying-Cheng Lai[3], and Celso Grebogi[4]

[1]*School of Systems Science, Beijing Normal University, Beijing, 100875, P. R. China*
[2]*Business School, University of Shanghai for Science and Technology, Shanghai 200093, China*
[3]*School of Electrical, Computer and Energy Engineering, Arizona State University, Tempe, Arizona 85287, USA*
[4]*Institute for Complex Systems and Mathematical Biology, Kings College, University of Aberdeen, Aberdeen AB24 3UE, UK*

Contents

1 Supplementary Note 1: The process of expanding payoff formula. 2

2 Supplementary Note 2: The process of calculating degrees of nodes 4

3 Supplementary Figures 5

4 Supplementary Table 11

5 Supplementary References 12

1 Supplementary Note 1: The process of expanding payoff formula.

To transform the relation between payoffs and strategies into the form $\mathbf{Y}_i = \Phi_i \cdot \mathbf{X}_i$, in which vector $\mathbf{X}_i$ includes the direct interactions and indirect interactions of player i with other players. The process which is like extract 'common factor' is shown as follows

$$\begin{aligned}
p_i &= \sum_{j=1}^{N} g_{ij}(e - c_i + \frac{b}{\sum_{l=1}^{N} g_{jl}} \sum_{l=1}^{N} g_{jl}c_l) \\
&= \begin{pmatrix} e - c_i + \frac{b}{\sum_{l=1}^{N} g_{1l}} \sum_{l=1}^{N} g_{1l}c_l \\ e - c_i + \frac{b}{\sum_{l=1}^{N} g_{2l}} \sum_{l=1}^{N} g_{2l}c_l \\ \vdots \\ e - c_i + \frac{b}{\sum_{l=1}^{N} g_{Nl}} \sum_{l=1}^{N} g_{Nl}c_l \end{pmatrix}^T \begin{pmatrix} g_{i1} \\ g_{i2} \\ \vdots \\ g_{iN} \end{pmatrix} \\
&= \begin{pmatrix} e - c_i \\ e - c_i \\ \vdots \\ e - c_i \end{pmatrix}^T \begin{pmatrix} g_{i1} \\ g_{i2} \\ \vdots \\ g_{iN} \end{pmatrix} + b \begin{pmatrix} \frac{\sum_{l=1}^{N} g_{1l}c_l}{\sum_{l=1}^{N} g_{1l}} \\ \frac{\sum_{l=1}^{N} g_{2l}c_l}{\sum_{l=1}^{N} g_{2l}} \\ \vdots \\ \frac{\sum_{l=1}^{N} g_{Nl}c_l}{\sum_{l=1}^{N} g_{Nl}} \end{pmatrix}^T \begin{pmatrix} g_{i1} \\ g_{i2} \\ \vdots \\ g_{iN} \end{pmatrix} \\
&= p^a + p^b,
\end{aligned} \tag{S1}$$

where p^a and p^b denote the first term and the second term on the right-hand side in Eq. (S1), respectively. Then we extend p^a and p^b as follows

$$\begin{aligned}
p^a &= \begin{pmatrix} e - c_i \\ e - c_i \\ \vdots \\ e - c_i \end{pmatrix}^T \begin{pmatrix} g_{i1} \\ g_{i2} \\ \vdots \\ g_{iN} \end{pmatrix} \\
&= \begin{pmatrix} e - c_i \\ e - c_i \\ \vdots \\ e - c_i \end{pmatrix}^T \begin{pmatrix} g_{11} & g_{21} & \cdots & g_{N1} \\ g_{12} & g_{22} & \cdots & g_{N2} \\ \vdots & \vdots & \ddots & \vdots \\ g_{1N} & g_{2N} & \cdots & g_{NN} \end{pmatrix} \times \\
&\quad \begin{pmatrix} \frac{1}{\sum_{l=1}^{N} g_{1l}} & 0 & \cdots & 0 \\ 0 & \frac{1}{\sum_{l=1}^{N} g_{2l}} & \cdots & 0 \\ \vdots & \vdots & \ddots & \vdots \\ 0 & 0 & \cdots & \frac{1}{\sum_{l=1}^{N} g_{Nl}} \end{pmatrix} \begin{pmatrix} g_{i1} \\ g_{i2} \\ \vdots \\ g_{iN} \end{pmatrix}
\end{aligned} \tag{S2}$$

$$
\begin{aligned}
p^b &= b \begin{pmatrix} \frac{\sum_{l=1}^N g_{1l}c_l}{\sum_{l=1}^N g_{1l}} \\ \frac{\sum_{l=1}^N g_{2l}c_l}{\sum_{l=1}^N g_{2l}} \\ \vdots \\ \frac{\sum_{l=1}^N g_{Nl}c_l}{\sum_{l=1}^N g_{Nl}} \end{pmatrix}^T \begin{pmatrix} g_{i1} \\ g_{i2} \\ \vdots \\ g_{iN} \end{pmatrix} \\
&= b \begin{pmatrix} \sum_{l=1}^N g_{1l}c_l \\ \sum_{l=1}^N g_{2l}c_l \\ \vdots \\ \sum_{l=1}^N g_{Nl}c_l \end{pmatrix}^T \begin{pmatrix} \frac{1}{\sum_{l=1}^N g_{1l}} & 0 & \cdots & 0 \\ 0 & \frac{1}{\sum_{l=1}^N g_{2l}} & \cdots & 0 \\ \vdots & \vdots & \ddots & \vdots \\ 0 & 0 & \cdots & \frac{1}{\sum_{l=1}^N g_{Nl}} \end{pmatrix} \begin{pmatrix} g_{i1} \\ g_{i2} \\ \vdots \\ g_{iN} \end{pmatrix} \\
&= b \begin{pmatrix} c_1 \\ c_2 \\ \vdots \\ c_N \end{pmatrix}^T \begin{pmatrix} g_{11} & g_{21} & \cdots & g_{N1} \\ g_{12} & g_{22} & \cdots & g_{N2} \\ \vdots & \vdots & \ddots & \vdots \\ g_{1N} & g_{2N} & \cdots & g_{NN} \end{pmatrix} \times \\
&\begin{pmatrix} \frac{1}{\sum_{l=1}^N g_{1l}} & 0 & \cdots & 0 \\ 0 & \frac{1}{\sum_{l=1}^N g_{2l}} & \cdots & 0 \\ \vdots & \vdots & \ddots & \vdots \\ 0 & 0 & \cdots & \frac{1}{\sum_{l=1}^N g_{Nl}} \end{pmatrix} \begin{pmatrix} g_{i1} \\ g_{i2} \\ \vdots \\ g_{iN} \end{pmatrix}.
\end{aligned} \tag{S3}
$$

So we can expand payoff formula of player i as

$$
\begin{aligned}
p_i &= p^a + p^b \\
&= \begin{pmatrix} bc_1 + e - c_i \\ bc_2 + e - c_i \\ \vdots \\ bc_N + e - c_i \end{pmatrix}^T \begin{pmatrix} g_{11} & g_{21} & \cdots & g_{N1} \\ g_{12} & g_{22} & \cdots & g_{N2} \\ \vdots & \vdots & \ddots & \vdots \\ g_{1N} & g_{2N} & \cdots & g_{NN} \end{pmatrix} \times \\
&\begin{pmatrix} \frac{1}{\sum_{l=1}^N g_{1l}} & 0 & \cdots & 0 \\ 0 & \frac{1}{\sum_{l=1}^N g_{2l}} & \cdots & 0 \\ \vdots & \vdots & \ddots & \vdots \\ 0 & 0 & \cdots & \frac{1}{\sum_{l=1}^N g_{Nl}} \end{pmatrix} \begin{pmatrix} g_{i1} \\ g_{i2} \\ \vdots \\ g_{iN} \end{pmatrix}
\end{aligned} \tag{S4}
$$

2 Supplementary Note 2: The process of calculating degrees of nodes

The vector $\mathbf{X}_i$ containing direct interactions and indirect interactions can be optimized via the Lasso. To extract adjacency matrix A from combined matrix C by virtue of similarity transformation and the linear squares method, we need to obtain degree of each node. Coincidentally, we can achieve degree of node i by summation of vector $\mathbf{X}_i$ as follows

$$\begin{aligned}
\mathbf{X}_i &= GD\mathbf{G}_i \\
&= \begin{pmatrix} g_{11} & g_{21} & \cdots & g_{N1} \\ g_{12} & g_{22} & \cdots & g_{N2} \\ \vdots & \vdots & \ddots & \vdots \\ g_{1N} & g_{2N} & \cdots & g_{NN} \end{pmatrix} \begin{pmatrix} \frac{1}{\sum_{l=1}^{N} g_{1l}} & 0 & \cdots & 0 \\ 0 & \frac{1}{\sum_{l=1}^{N} g_{2l}} & \cdots & 0 \\ \vdots & \vdots & \ddots & \vdots \\ 0 & 0 & \cdots & \frac{1}{\sum_{l=1}^{N} g_{Nl}} \end{pmatrix} \begin{pmatrix} g_{i1} \\ g_{i2} \\ \vdots \\ g_{iN} \end{pmatrix} \\
&= \begin{pmatrix} \frac{g_{11}}{\sum_{l=1}^{N} g_{1l}} & \frac{g_{21}}{\sum_{l=1}^{N} g_{2l}} & \cdots & \frac{g_{N1}}{\sum_{j=1}^{N} g_{Nl}} \\ \frac{g_{12}}{\sum_{l=1}^{N} g_{1l}} & \frac{g_{22}}{\sum_{l=1}^{N} g_{2l}} & \cdots & \frac{g_{N2}}{\sum_{j=1}^{N} g_{Nl}} \\ \vdots & \vdots & \ddots & \vdots \\ \frac{g_{1N}}{\sum_{l=1}^{N} g_{1l}} & \frac{g_{2N}}{\sum_{l=1}^{N} g_{2l}} & \cdots & \frac{g_{NN}}{\sum_{j=1}^{N} g_{Nl}} \end{pmatrix} \begin{pmatrix} g_{i1} \\ g_{i2} \\ \vdots \\ g_{iN} \end{pmatrix}
\end{aligned} \tag{S5}$$

$$\begin{aligned}
\sum \mathbf{X}_i &= \frac{g_{11}g_{i1}}{\sum_{l=1}^{N} g_{1l}} + \frac{g_{21}g_{i2}}{\sum_{l=1}^{N} g_{2l}} + \cdots + \frac{g_{N1}g_{iN}}{\sum_{l=1}^{N} g_{Nl}} \\
&+ \frac{g_{12}g_{i1}}{\sum_{l=1}^{N} g_{1l}} + \frac{g_{22}g_{i2}}{\sum_{l=1}^{N} g_{2l}} + \cdots + \frac{g_{N2}g_{iN}}{\sum_{l=1}^{N} g_{Nl}} + \cdots \\
&+ \frac{g_{1N}g_{i1}}{\sum_{l=1}^{N} g_{1l}} + \frac{g_{2N}g_{i2}}{\sum_{l=1}^{N} g_{2l}} + \cdots + \frac{g_{NN}g_{iN}}{\sum_{l=1}^{N} g_{Nl}} \\
&= \frac{g_{11}g_{i1} + g_{12}g_{i1} + \cdots + g_{1N}g_{i1}}{\sum_{l=1}^{N} g_{1l}} + \frac{g_{21}g_{i2} + g_{22}g_{i2} + \cdots + g_{2N}g_{i1}}{\sum_{l=1}^{N} g_{2l}} \\
&+ \cdots + \frac{g_{N1}g_{iN} + g_{N2}g_{iN} + \cdots + g_{NN}g_{iN}}{\sum_{l=1}^{N} g_{Nl}} \\
&= \frac{g_{i1}\sum_{l=1}^{N} g_{1l}}{\sum_{l=1}^{N} g_{1l}} + \frac{g_{i2}\sum_{l=1}^{N} g_{2l}}{\sum_{l=1}^{N} g_{2l}} + \cdots + \frac{g_{iN}\sum_{l=1}^{N} g_{Nl}}{\sum_{l=1}^{N} g_{Nl}} \\
&= g_{i1} + g_{i2} + \cdots + g_{iN} \\
&= d_i
\end{aligned} \tag{S6}$$

Thus, we can derive the degree of each node from $k_i = d_i - 1 = \sum \mathbf{X}_i - 1$, where $\mathbf{X}_i = \mathbf{C}_i$

3 Supplementary Figures

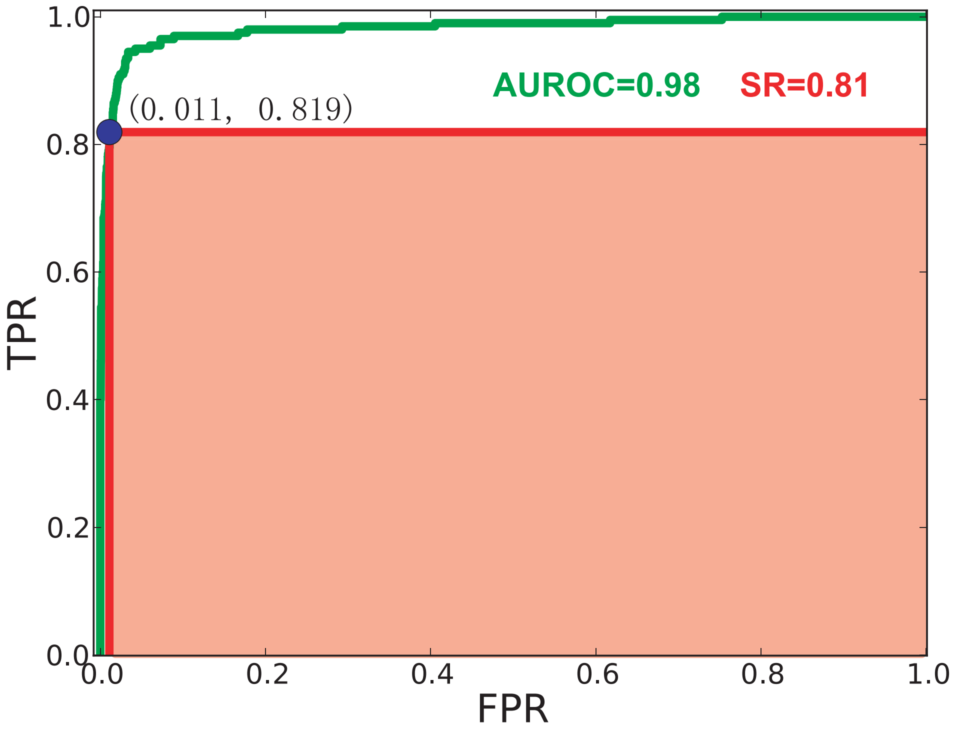

Supplementary Figure S1: Illustration of the relation between the receiver operating characteristic curve (AUROC) and the success rate (SR). True positive rate (TPR) vs false positive rate (FPR) for $\text{Data} = 0.3$ on Watts-Strogatz (WS) small-world network with network size $N = 100$, average degree $\langle k \rangle = 4$, and rewiring probability 0.3, and the corresponding AUROC is 0.98 (the area under the green curve). Because the degree of each node can be inferred from the formula $k_i = d_i - 1 = \sum \mathbf{X}_i - 1$, we can set the top k_i candidates as positive links, and calculate the corresponding values of TPR(k_i) and FPR(k_i) (the blue point). Then we define the success rate (SR) to be $\text{SR} = \text{TPR}(k_i) \times \text{TNR}(k_i)$, where $\text{TNR} = 1 - \text{FPR}$ is the true negative rate. In this case SR of reconstruction is 0.81 for $\text{Data} = 0.3$ (the area of red rectangle). Note that the blue point is in the receiver operating characteristic curve, SR can be regarded the area of TPR versus FPR with given threshold. When SR reaches 1, or equivalently, TPR and FPR reach 1 and 0, respectively, AUROC will be unit, indicating the network is fully reconstructed.

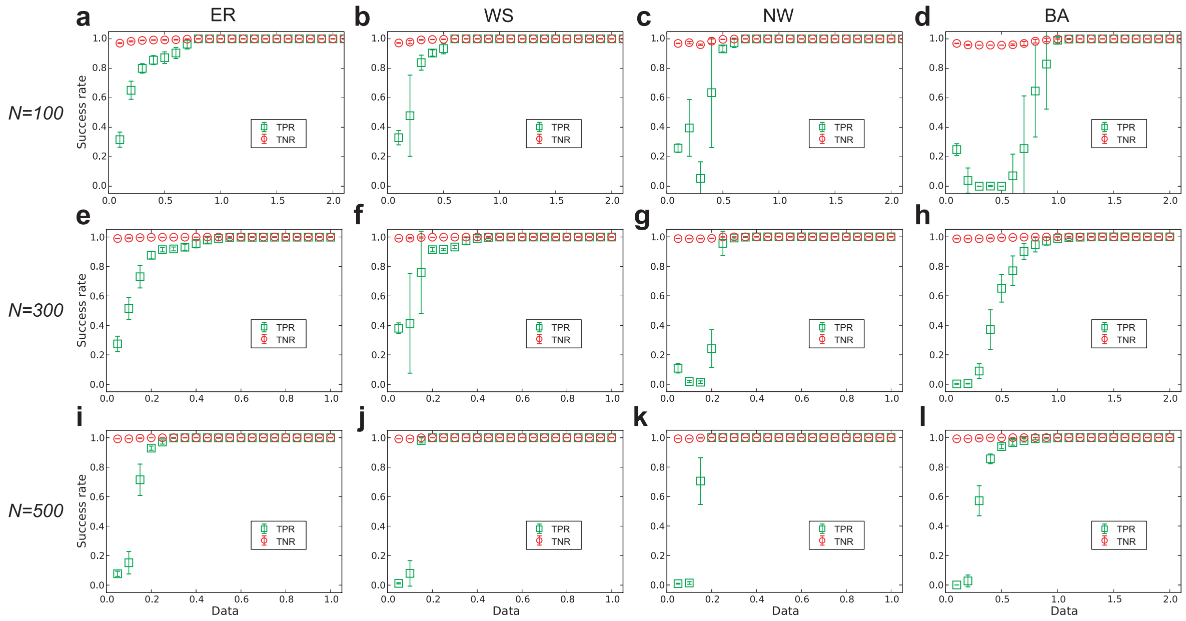

Supplementary Figure S2: Success rate of inferring four artificial models in different network size. Success rate of inferring Erdös-Rényi (ER) random networks, Watts-Strogatz (WS) small-world networks, Newman-Watts (NW) small-world networks and Barabási-Albert (BA) scale-free networks at different network size, based on time series obtained from evolutionary networked PGGs. The true positive rate (TPR) is defined as the ratio of the number of successfully inferred links to the number of existent links for the entire network, and the true negative rate (TNR) is similar defined for zero elements in the adjacency matrix. (**a**-**d**) network size $N = 100$, (**e**-**h**) network size $N = 300$, and (**i**-**l**) network size $N = 500$. The average degree $\langle k \rangle = 4$. Each data point is obtained by averaging over 10 independent realizations. The error bars denote the standard deviations. Rewiring probability of WS networks is 0.3. For the NW networks, each node in the ring is connected with its 2 nearest neighbors and the probability of adding a new edge for each edge is 1. For each type of network, as network size increases, the amount of data required to reconstruct networks accurately decreases, especially for homogeneous networks, e.g., ER, WS and NW networks.

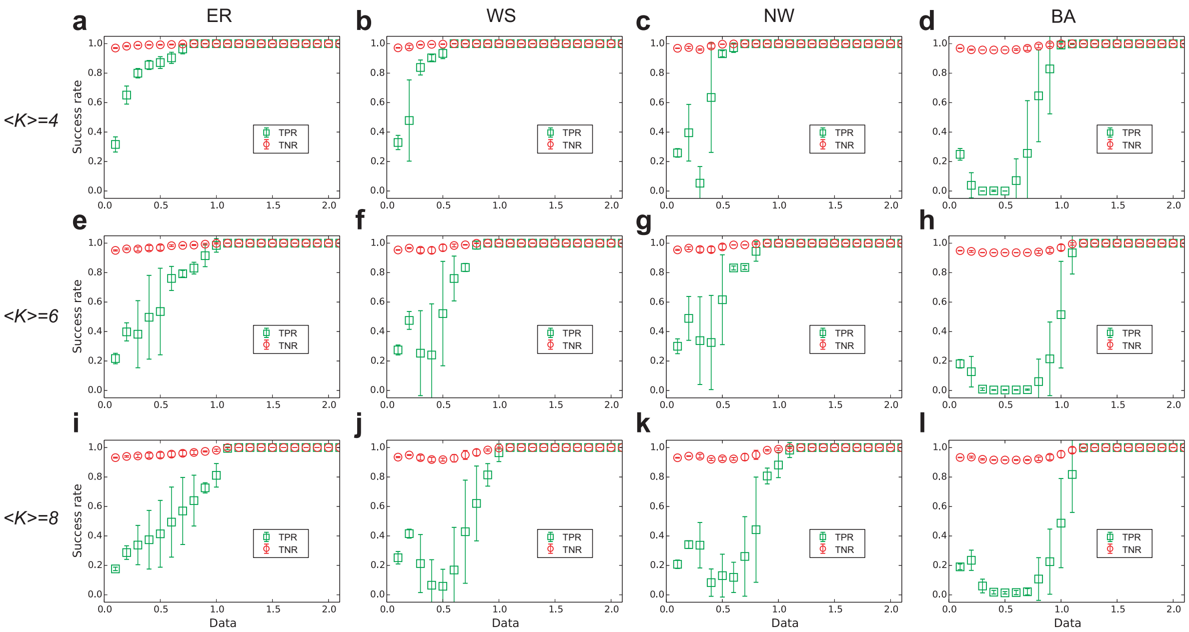

Supplementary Figure S3: Success rate of inferring four artificial models in different average degree. Success rate of inferring ER, WS, NW and BA networks at different average degree, based on time series obtained from evolutionary networked PGGs. TPR and TNR are the same as in Supplementary Fig. S2. (**a**-**d**) average degree $\langle k \rangle = 4$, (**e**-**h**) average degree $\langle k \rangle = 6$, and (**i**-**l**) average degree $\langle k \rangle = 8$. The network size N is 100. Each data point is obtained by averaging over 10 independent realizations. The error bars denote the standard deviations. Rewiring probability of WS networks is 0.3. For NW networks with average degree $\langle k \rangle = 4$, each node in the ring is connected with its 2 nearest neighbors and the probability of adding a new edge for each edge is 1; for NW networks with average degree $\langle k \rangle = 6$, each node in the ring is connected with its 4 nearest neighbors and the probability of adding a new edge for each edge is 0.5; for NW networks with average degree $\langle k \rangle = 8$, each node in the ring is connected with its 4 nearest neighbors and the probability of adding a new edge for each edge is 1. For each type of network, as average degree increases, the amount of data required to reconstruct networks accurately also increases.

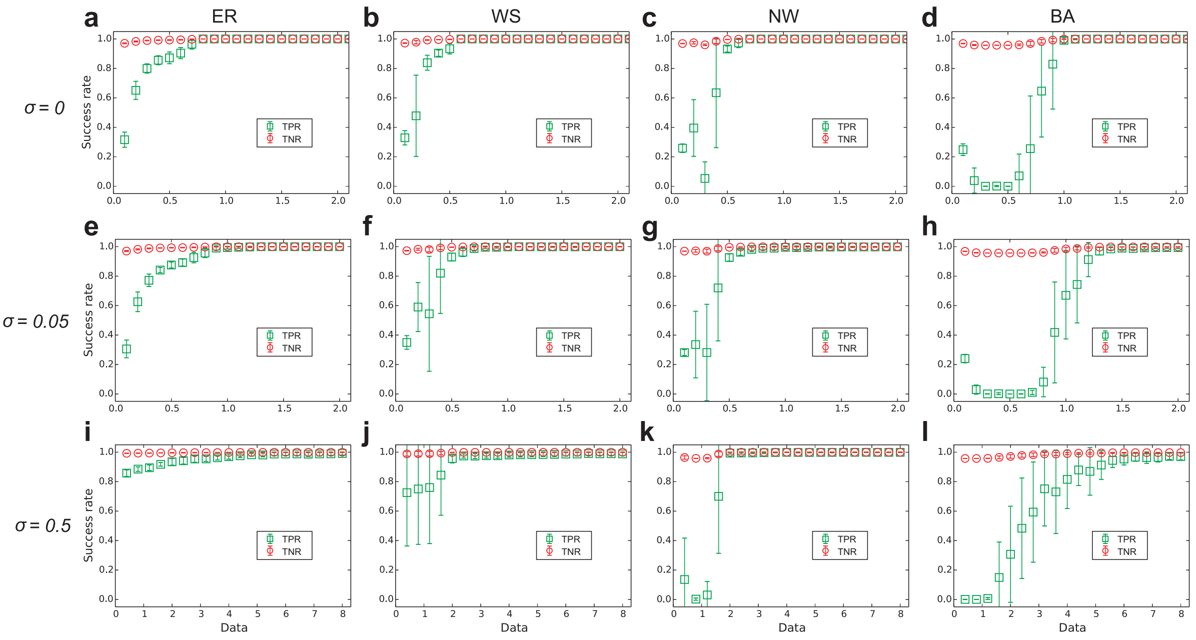

Supplementary Figure S4: Success rate of inferring four artificial models in different Gaussian noise. Success rate of inferring ER, WS, NW and BA networks based on time series obtained from evolutionary networked PGGs with adding different Gaussian noise to payoffs. TPR and TNR are the same as in Supplementary Fig. S2. (**a**-**d**) there are no noise, (**e**-**h**) the distribution of noise is $\mathcal{N}(0, 0.05^2)$, and (**i**-**l**) the distribution of noise is $\mathcal{N}(0, 0.5^2)$. The network size N is 100. The average degree $\langle k \rangle = 4$. Each data point is obtained by averaging over 10 independent realizations. The error bars denote the standard deviations. Rewiring probability of WS networks is 0.3. For the NW networks, each node in the ring is connected with its 2 nearest neighbors and the probability of adding a new edge for each edge is 1. For each type of network, as Gaussian noise increases, the amount of data required to reconstruct networks accurately also increases, especially for heterogeneous networks, e.g., BA networks.

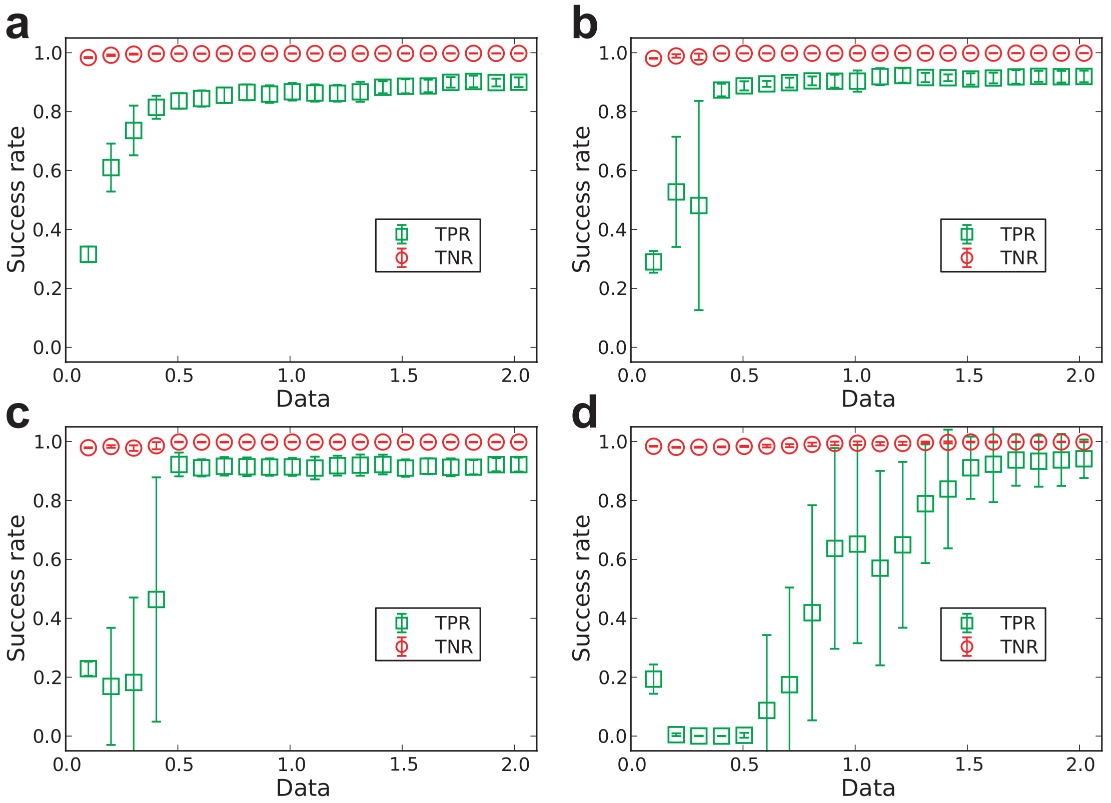

Supplementary Figure S5: The success rate of reconstructing different networks with a hidden node in each network. The success rate of inferring (**a**) ER, (**b**) WS, (**c**) NW and (**d**) BA networks with a hidden node in each network based on time series obtained from evolutionary networked PGGs. TPR and TNR are the same as in Supplementary Fig. S2. The network size N is 100. The average degree $\langle k \rangle = 4$. Each data point is obtained by averaging over 10 independent realizations. The error bars denote the standard deviations. Rewiring probability of WS networks is 0.3. For the NW networks, each node in the ring is connected with its 2 nearest neighbors and the probability of adding a new edge for each edge is 1.

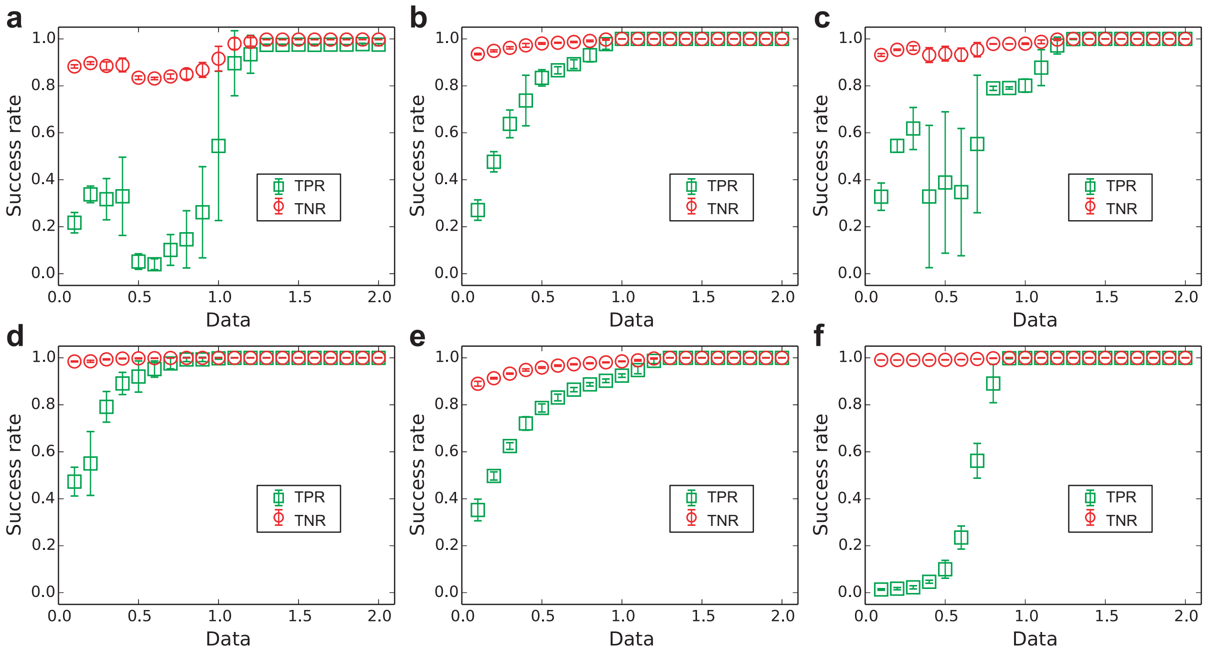

Supplementary Figure S6: Success rate of inferring six real social networks. Success rate of inferring the Karate network (**a**), the Dolphins network (**b**), the Football network (**c**), the Santa Fe network (**d**), the Jazz network (**e**) and the Email network (**f**) based on time series obtained from evolutionary networked PGGs. TPR and TNR are the same as in Fig. S2. Each data point is obtained by averaging over 10 independent realizations. The error bars denote the standard deviations. Details of the real social networks are shown in Supplementary Table S1.

4 Supplementary Table

In the main text, we test the performance of our reconstruction framework with several real social networks. The detail of real social networks are shown in the Supplementary Table S1.

Supplementary Table S1: Summary of the real social networks analyzed in the paper. Here N is network sizes, L is the number of links and $\langle k \rangle$ denotes average degree of a network. The data can be found in relevant references.

Name	N	L	$\langle k \rangle$	Description
Karate [1]	34	78	4.6	Network of friendship in a karate club
Dolphins [2]	62	159	5.1	Frequent associations between 62 dolphins
Football [3]	115	613	10.7	Network of American college football game
Santa Fe [3]	118	199	3.4	Scientific collaboration network of the Santa Fe Institute
Jazz [4]	198	2742	27.7	Network of jazz musicians
Email [5]	1133	5451	9.6	Network of email interchanges

5 Supplementary References

[S1]. Zachary, W. W. An information flow model for conflict and fission in small groups. *J. Anthropol. Res.* 452–473 (1977).

[S2]. Lusseau, D. *et al.* The bottlenose dolphin community of doubtful sound features a large proportion of long-lasting associations. *Behav. Ecol. Sociobiol.* **54**, 396–405 (2003).

[S3]. Girvan, M. & Newman, M. E. Community structure in social and biological networks. *Proc. Natl. Acad. Sci. USA* **99**, 7821–7826 (2002).

[S4]. Gleiser, P. M. & Danon, L. Community structure in jazz. *Adv. Complex Syst.* **6**, 565–573 (2003).

[S5]. Guimera, R., Danon, L., Diaz-Guilera, A., Giralt, F. & Arenas, A. Self-similar community structure in a network of human interactions. *Phys. Rev. E* **68**, 065103 (2003).
